# Supplementary material for: Personalised pose estimation from single-plane moving fluoroscope images using deep convolutional neural networks
Source: PLoS One. 2022 Jun 24;17(6):e0270596. doi: 10.1371/journal.pone.0270596 (PMC9231734; doi:10.1371/journal.pone.0270596)
Supplement: Attachment — Submitted filename: Response to Reviewers2.docx [file pone.0270596.s001.docx]

**Personalised pose estimation from single-plane moving fluoroscope images using deep convolutional neural networks**

by Vogl Florian, Schütz Pascal, Postolka Barbara, List Renate, William Taylor

We would like to thank the Reviewers for their critical feedback to our manuscript, which has helped to improve our manuscript - a brief point-by-point response to Reviewers’ comments is presented below. Wherever possible, we have included the modified text or added text passage for your convenience highlighted in red.

## Reviewer #1:

The paper is well-motivated and the introduction of the Deep Auto-Match Network (DAMN) is novel. However, there are some concerns that should be addressed:

1. DAMN consists of two parts and contains various loss functions, and it is recommended to perform ablation experiments to show the role of each part. More explanation and intuition on DAMN are needed.

DAMN indeed consists of two parts, one segmentation network and a regression network to predict the pose from this segmentation. Our goal was to build the simplest network architecture to achieve personalised 2D-3D pose estimation of implants based on standard models from computer vision. In fact, we used an ablation-procedure, starting from a 1) a generic regression network, 2) individualised regression networks, 3) generic segmentation + generic regression, 4) generic segmentation + personalised regression networks, to 5) include recursions to build an iterative process. However, we were unable to achieve any success with models 1-3, which most likely is due to the fact that the implant coordinate systems cannot be standardized. We thus decided to just report 4) and 5) in our manuscript.

While we have intentionally chosen the simplest standard approaches, changes to the models themselves (e.g. using a different segmentation network), changes to the loss function for either segmentation or regression networks, optimisers or training regime, might all affect performance and could be investigated further. However, considering the exceptional computational requirements (multiple weeks per investigation) we have deemed it more important to first address the more fundamental problems of a) dealing with occlusions, b) extending the method to work with healthy bone, which we are currently working on. We still hope that our approach and the complete codebase will give other researchers the tools to directly investigate such questions and optimise this method for their own data.

2. It is recommended that the time complexity should be discussed and analyzed in detail.

We have added a discussion of the required training time.

Using this procedure, we first trained one synthnet to completion for about five days, and then froze the first quarter of the network before training the individualised networks for each of the $53$ femoral/tibial implant combinations, which took about one day for each synthnet on a Nvidia GTX2080Ti GPU. Freezing the input layers of the synthnet ensured that the input features remained consistent between different synthnets, which was required as the synthnet part was swapped out during the training process of the shadownet in the next step.

(…)

Training of the shadownet took about five days on a Nvidia GTX2080 Ti GPU, with improvements slowing down considerably after about three days. Overall, the training of the complete, personalised DAMN model took about 63 GPU days, which we parallelised using multiple GPUs. After the initial training, any new subject will only require a new personalised synthnet, which can be trained in 1 day using transfer-learning.

(…)

However, use of these larger images substantially increases the size of the model and thus the required memory and training time by a factor of four. Considering the substantial training time (for a Nvidia GTX 2080 Ti: 5 days for the initial synthnet, 1 day for each implant combination, 5 days for the shadownet) this complexity increase seems infeasible without making considerable improvements to either the model or the training process.

In addition, the parameters of the DAMN should also be analyzed numerically.

We are unsure what you mean by analyzing the parameters of the DAMN numerically. We performed some training runs with different values for parameters such as the optimiser or learning rate, but performing full training runs with e.g. different convolution kernels it out of our capabilities due to the required training times. Below you can find an example on how we found the learning rate for the synthnet by evaluating the loss after the early training process.

3. Since some data is difficult to obtain, it is worth considering whether the performance can be improved by generating data. For example, some work similar to [1] may have some inspiration.

[1] Gong, An, Xinjie Yao, and Wei Lin. "Dermoscopy image classification based on StyleGANs and decision fusion." Ieee Access 8 (2020): 70640-70650.

We are grateful for this suggestion – we have included a discussion in our manuscript.

In fact, we are already using generated data, as we are generating synthetic renderings of the personalised implant geometries in different poses to train the synthnet. We have added the following section to explain our reasoning.

First, we pretrain the synthnet on synthetically rendered, binarized (shadow) images of each individual femoral/tibial implant combination. Because the parameter space of the two implant poses is so high-dimensional, using purely random poses would lead to a lot of non-sensical situations, such as when the femur and tibia are impossibly overlapping or impossibly far apart considering the anatomical constraints, or would lead to poses that are irrelevant for activities of daily living (e.g. upside-down). To keep the model focussed on the task and the computational time low, it is thus important to focus on creating implant poses that are close to the ones arising in activities of daily living. Towards this goal, we perturb poses from our dataset of activities of daily living using Gaussian noise for each of the 18 pose components to create realistic poses close to the original kinematics.

4. There are several works on the combination of deep learning and medicine, and it is suggested to compare with some state-of-the-art methods. In addition, this work discussed x-ray CT and its strengths and disadvantages, it is time-intensive, expensive, and operator-dependent. In fact, in the field of porous media/materials, the same problem exists with the use of X-CT, see, Fractals-Complex geometry patterns and scaling in nature and society, 2018, 26(2): 1840003. https://doi.org/10.1142/S0218348X18400030, and Physics of Fluids, 2021, 33(3): 032013. https://doi.org/10.1063/5.0042606. It is suggested that the authors refer to these recent works to strengthen the technical context of this ms, support the point of view about X-CT and attract more readers.

We have included a paragraph discussing the similarities and differences, as well as the possible synergies between implant radiographs and Xray-CT of objects with less contrast.

Our approach can further be extended to estimate the poses of natural bone instead of implants. Assuming that the bone geometry is known (e.g. from CT), our approach can be used with no further modifications. Indeed, for certain applications, our approach of a segmentation and a personalized regression network can also be used to estimate poses from CT-data, thus opening opportunities in other fields using CT or µ-CT, such as research on porous media [33-35]. While our approach is easily extensible to deal with volumetric data (instead of 2D images) by using standard 3D-networks, the main limitation remains the requirement that the target object’s geometry must be known to personalise the regression network. Because implants have high absorption and thus appear as nearly uniform shapes in the x-ray image, our approach binarizes the segmentation and regression networks. For estimating the poses of structures with lower absorption that show more internal structure on x-ray images (e.g. bone), one could replace our binary segmentation and regression networks with greyscale versions to consider this additional information. However, it is likely that this version would be more susceptible to noise or occlusion effects, so it remains to be investigated whether a binarized or grey-scale approach is advantageous for estimating the poses of natural bone.

Reviewer #2:

Paper deals with hot topic. It has scientific novelty and great practical value.

Paper has a logical structure all necessary sections. The paper is technically sound. The experimental section is good.

The proposed approach is logical, results are clear.

Suggestions:

(1) Can authors explain more about 6D representation of predicted rotation and automated method to perform 2D-3D pose estimation?

Rotation can be represented in different ways and have different advantages and disadvantages – both fundamentally or with respect to deep learning. For example, Euler-Angles cannot be interpolated easily and suffer from Gimbals lock, or quaternions, whose double-coverage (two quaternions representing the same 3D rotation) can cause problems for the optimisation procedure as it is unclear which of the two quaternions to optimise towards. The work referenced in the manuscript (Zhou Y, et al. On the Continuity of Rotation Representations in Neural Networks. 2019 IEEE/CVF Conference on Computer Vision and Pattern Recognition (CVPR). 2018) investigated different representations and found the 6D representation, or 6 entries from the 3x3 rotation matrix, to be the best representation for deep learning. We considered adding additional information on this topic to the manuscript, but were afraid it would be too technical of a point – please let us know if you would find such information helpful, and we will happily add an explanatory paragraph to the manuscript.

(2) How the quality of the 3D implant information effects performance in pose estimation task?

This is an excellent observation, as the 3D implant information is indeed a critical part of the pose estimation process – if the algorithm does not know what to look for, how should it be able to find it? This issue is even more pronounced when dealing with natural bone, as the 3D geometry is usually acquired by CT, which has limited resolution and depends on the segmentation method. The effect depends on the concrete deviations between the actual object and the 3D information. For example, if the real implant is smaller than the target 3D geometry the out-of-plane error will increase, as the algorithm will move the prediction farther away to decrease its apparent size. We made an attempt of discussing such issues in the following paragraph:

Our approach requires that the 3D information of the target object is accurately known – if the algorithm does not know what do look for, how should it be able to find and estimate its pose? While we had no way of measuring the implant geometries directly, manufacturing discrepancies from the implant designs (which were available) are assumed to be negligible compared to other error sources. If the 3D geometry of the target object is uncertain (e.g. when estimating the pose of natural bone, which is often acquired by CT, which has a limited resolution), this effect will be more pronounced and should be investigated. Here, different discrepancies (scale, loss of detail, slight geometric changes of one part of the outline) could be investigated with respect to their effect on the estimation result and compared to the results of the gold standard using the modified geometry. While this process seems prohibitively time- and cost-intensive when using manual registration as gold standard, an automated approach using mechanical precision devices could provide a practical implementation.

(3) In common we uses high resolution images in deep learning algorithms. What is your approach? If you are not using high resolution images then why?

Our original images are 1000 x 1000 pixels, limited by the image intensifier and the CCD camera. For our work, we downscale the images to 512x512 pixel images to reduce the memory footprint and the computational time. While using the higher resolution images is very likely to indeed improve performance, it would require significant investment in terms of hardware of technology to use the larger models while still keeping training times low enough to allow for prototyping and evaluation. We have thus decided to first tackle other issues, such as dealing with low contrast or occlusions or an extension to natural bone using greyscale information, which is work in progress. We have also added the following paragraph:

For example, the in-plane translation performance of our approach is about \SI{0.75}{\mm}, which corresponds to about one to two pixels for the downsampled 512px images. Considering this resolution limit, it is likely that the performance of our approach could be further improved by using the original 1000px images. However, use of these larger images substantially increases the size of the model and thus the required memory and training time by a factor of four. Considering the substantial training time (for a Nvidia GTX 2080 Ti: 5 days for the initial synthnet, 1 day for each implant combination, 5 days for the shadownet) this complexity increase seems impractical without making considerable improvements to either the model or the training process - particularly during development (e.g. dealing with occlusions) where rapid prototyping and evaluation is necessary.

(4) If it is not restricted in journal norms then one Conclusion Section will help interested authors to attract readers to go read this research work.

We have now provided a conclusion to help readers quickly decide whether they are interested in reading the full paper.

This work presented a deep-learning based approach to estimate joint poses from fluoroscopic images, which is personalised to each subject without any additional manual pose registration. Depending on the required performance, our approach can be used directly to predict poses to about 0.75mm (in-plane translation) and 2° (all Euler-angle rotations), and can easily be extended to natural bone, multi-plane fluoroscopy, or even CT, assuming that the target object’s geometry is known. Alternatively, our approach can be combined with pose refinement algorithms [citations] by providing approximate starting poses in a fully automated manner, thus avoiding the laborious and expensive task of manually estimating the poses for each image.
